# A Facile Approach towards Fluorescent Nanogels with AIE-Active Spacers

**DOI:** 10.3390/polym10070722

**Published:** 2018-07-01

**Authors:** Meiran Feng, Laiping Fang, Fujun Guan, Siying Huang, Yinwei Cheng, Yancui Liang, Hefeng Zhang

**Affiliations:** Department of Chemistry and Key Laboratory for Preparation and Application of Ordered Structural Materials of Guangdong Province, Shantou University, Shantou 515063, China; 17mrfeng@stu.edu.cn (M.F.); 17lpfang@stu.edu.cn (L.F.); 16fjguan@stu.edu.cn (F.G.); 16syhuang3@stu.edu.cn (S.H.); ywcheng@stu.edu.cn (Y.C.); 16ycliang@stu.edu.cn (Y.L.)

**Keywords:** star-shaped polymeric precursor, fluorescent nanogel, AIE-active spacer

## Abstract

A facile and efficient approach for design and synthesis of organic fluorescent nanogels has been developed by using a pre-synthesized polymeric precursor. This strategy is achieved by two key steps: (i) precise synthesis of core–shell star-shaped block copolymers with crosslinkable AIEgen-precursor (AIEgen: aggregation induced emission luminogen) as pending groups on the inner blocks; (ii) gelation of the inner blocks by coupling the AIEgen-precursor moieties to generate AIE-active spacers, and thus, fluorescent nanogel. By using this strategy, a series of star-shaped block copolymers with benzophenone groups pending on the inner blocks were synthesized by grafting from a hexafunctional initiator through atom transfer radical copolymerization (ATRP) of 4-benzoylphenyl methacrylate (BPMA) or 2-(4-benzoylphenoxy)ethyl methacrylate (BPOEMA) with methyl methacrylate (MMA) and *tert*-butyldimethylsilyl-protected 2-hydroxyethyl methacrylate (ProHEMA) followed by a sequential ATRP to grow PMMA or PProHEMA. The pendent benzophenone groups were coupled by McMurry reaction to generate tetraphenylethylene (TPE) groups which served as AIE-active spacers, affording a fluorescent nanogel. The nanogel showed strong emission not only at aggregated state but also in dilute solution due to the strongly restricted inter- and intramolecular movement of TPE moiety in the crosslinked polymeric network. The nanogel has been used as a fluorescent macromolecular additive to fabricate fluorescent film.

## 1. Introduction

In comparison to their inorganic counterparts, including fluorescent nanoparticles and semiconductor quantum dots, organic luminescent nanomaterials have attracted continuous attention, due to their excellent processability, flexibility, low toxicity, degradability, high biocompatibility and structural versatility [1,2]. Up to now, various organic fluorescent nanomaterials have been developed, including fluorescent protein [3,4], dye-doped non-emissive micelles [5], assemblies of amphiphilic dyes [6], or polymers functionalized with fluorescent side or terminal groups [7], and polymer nanoparticle encapsulating dyes [8], etc. and found applications in sensors [9,10], cell thermometers [11,12], bio-imaging [13], drug delivery [13,14], and more.

Among the organic nanomaterials, nanogels constructed by covalently crosslinked polymeric network incorporating fluorophore groups are reasonably supposed to have higher chemical and mechanical stability [12,15] than those constructed through van der Waals forces [1]. For nanogel synthesis, several methods have been developed, including crosslinking the pre-synthesized linear block copolymers by coupling their functional side groups [16,17,18,19], initiating difunctional monomer by using a polymeric macroinitiator, and polymerizing difunctional monomers in a nano-sized emulsion [20,21,22], etc. Recently, the utility of core–shell star-shaped block copolymers as precursors has emerged as a powerful strategy to construct nanoobjects with high controllability over the size and uniformity [23,24]. The living polymerization techniques promised well-defined and tunable structures of the star-shaped polymers, and thus, high controllability over the size, uniformity, and peripheral functional groups/blocks of the resultant nanogel, which were difficult to achieve by using conventional strategies. Rzayev’s group has successfully synthesized a series of tube-shaped nanogels with well-defined and tunable structures [25,26]. Lin et al. has employed a 21-arm star-shaped block copolymer of PCl_core_-*b*-PAzoSt_corona_ (PCL: poly(ε-carpolactone), PAzoSt: poly(4-azomethylstyrene)) as a precursor to synthesize a hollow nanogel by crosslinking the pendent azide groups on the outer blocks [27].

On the other hand, the conjugated dyes with planar and rigid structures, including rhodamines [28,29], fluoresceins [30], and naphthalimides [31,32], as well as their derivatives [33], have been widely used as fluorophores for a long period [8,34]. However, the brightness of these kinds of luminogens are strongly limited by their aggregation-caused quenching (ACQ) characteristics, such that the emission is significantly weakened or even quenched at high concentration. Consequently, the dyes can only be used in a dilute solution, leading to limitations including difficulty of further improvement in brightness, low photostability, and short life-time due to the small amount of fluorophores [8]. Different from the regular ACQ-type luminogens, aggregation-induced emission luminogens (AIEgen) are not emissive in dilute solution, but show strong emission at aggregate state because of highly restricted intramolecular rotation and vibration [35]. Since being developed by Tang’s group in 2001 [36], AIE strategy has opened a new horizon to luminescent and photoelectric materials design, witnessed by the successful synthesis of various novel AIEgens and their derivatives [37,38,39,40]. The emissions of current AIEgens have covered a wide spectrum range from blue to red or even to infrared, with higher photo and chemical stability, brightness, and longer lifetime, and found applications in bio-imaging [41,42,43,44,45,46,47], cancer theranostics [48,49], sensors [50,51,52,53], organic light emission diodes (OLED) [54,55,56,57], and other photovoltaic devices [58,59,60]. Benefiting from its intrinsic emissive characteristics, the AIEgen unit is an ideal fluorophore and crosslinker for constructing fluorescent organic nanogels in which the AIEgen moiety is fixed in a limited space, leading to high local concentration with highly restricted intramolecular movability, due to the huge steric hindrance of the polymeric network. For example, Wei’s group has synthesized fluorescent nanogels by copolymerizing AIE-active difunctional vinyl monomer with a zwitterionic methacrylate monomer through reversible addition–fragmentation transfer (RAFT) polymerization [61]. Zhang and Hadjichistidis et al. have employed a pre-synthesized linear 2-bromoisobutyrate terminated polyethylene as a macroinitiator to polymerize a TPE-contained (TPE: tetraphenylethylene) difunctional monomer leading to AIE-active fluorescent nanogels [62].

Inspired by the efficiency of polymeric precursor strategy, controllability of living polymerization techniques over the size and uniformity of star-shaped polymer and the specific emission nature of AIEgens at aggregate state, herein, we propose a facile access to organic fluorescent nanogels in which a pre-synthesized core–shell star-shaped block copolymer is employed as a precursor. After readily coupling the pendent benzophenone groups in the inner block, the TPE groups are generated, and served as AIE-active spacers leading to formation of polymeric network, and thus, fluorescent nanogels. The resultant nanogel is strongly emissive even at a dilute solution, due to the high local concentration and restricted structure of TPE groups.

## 2. Materials and Methods

### 2.1. Regents and Instruments

Methacryloyl chloride (95%, stabilized by 200 ppm MEHQ, Aladdin, Shanghai, China), 4-hydroxybenzophenone (98%, Aladdin, Shanghai, China), 2-bromoethanol (95%, Aladdin, Shanghai, China), potassium iodide (99%, Aladdin, Shanghai, China), dipentaerythritol (90%, Macklin, Shanghai, China), Titanium tetrachloride (99%, Energy Chemical, Shanghai, China), CuBr (99%, Macklin), *N*,*N*,*N*′,*N*″,*N*‴-pentamethyldiethylenetriamine (98%, Macklin, Shanghai, China) (PMDETA), anisole (99%, Aladdin, Shanghai, China), tetraphenylethylene (98%, TCI, Japan) (TPE), 2-hydroxyethyl methacrylate (HEMA) (99%, stabilized by 200 ppm MEHQ, Macklin, Shanghai, China) and *tert*-butyldimethylsilyl chloride (TBDMSCl) (99%, Energy Chemical, Shanghai, China) were used as received. Methyl methacrylate (99%, Macklin, Shanghai, China) and triethylamine (TEA) (99%, Aladdin, Shanghai, China) were distilled from CaH_2_ (95%, Aladdin, Shanghai, China) before use. All the solvents were purchased from Aladdin and were distilled from CaH_2_ before use.

Gel permeation chromatography analyses (GPC) were performed on a Wyatt liquid chromatography system with three identical columns (5 μm, 10 μm, 10 μm) equipped with a multiangle laser light scattering detector (MALLS) (Wyatt DAWN HELEOS LS II, Wyatt Technology Corp., Santa Barbara, USA) (λ = 658 nm), viscometer (Wyatt ViscoStar III, Wyatt Technology Corp., Santa Barbara, USA) and refractometer (Wyatt Optilab T-rEX, Wyatt Technology Corp., Santa Barbara, USA) (λ = 690 nm). THF (35 °C, 1 mL/min) was used as the mobile phase. ASTRA software (Wyatt Technology Corp., Wyatt Technology Corp., Santa Barbara, USA) was used to process the data. ^1^H and ^13^C NMR spectra were measured on a Bruker AVANCE II 400 spectrometer operating at 400 MHz (Bruker, Karlsruhe, Germany). Deuterated chloroform (CDCl_3_) was used as solvent with 1% TMS as an internal reference. Atom force microscopy (AFM) was performed on Bruker Scanning Probe Microscope (Bruker, Karlsruhe, Germany) by using a clean silica wafer as substrate. The dilute nanogel solution was spin-coated onto the clean silica wafer by (3000 r/min for 1 min) and was visualized by using a tapping mode. Fourier-transform infrared (FTIR) spectra were recorded on a Thermo Nicolet IR200 FT-IR spectrometer (Thermo Fisher Scientific China Co. Ltd, Shanghai, China) in the solid state in KBr. Fluorescent emission and excitation spectra were recorded on a Lengguang F98 fluorescence spectrophotometer (Lengguang Technology Co. Ltd., Shanghai, China). Dynamic light scattering (DLS) measurement was performed on Zetasizer Nano ZS90 (Malvern Panalytical Shanghai Ltd., Shanghai, China). Quantum yield of the nanogel solution (1 mg/mL) was measured in a stoppered quartz cuvette (1 cm path length) on a HORIBA JOBIN YVON Fluoromax-4P spectrofluorometer (Jobin Yvon, Paris, France) by using an integrated sphere.

### 2.2. Synthesis of Hexafunctional Initiator

The hexafunctional initiator was synthesized by esterifying dipentaerythritol with 2-bromo-2-methylpropanoyl bromide. Dipentaerythritol (1.60 g, 6.3 mmol, 1.0 equiv), TEA (11.00 mL, 75.4 mmol, 12.0 equiv) and DCM (100 mL) were added to a flask. After being cooled to 0 °C, 2-bromo-2-methylpropanoyl bromide (7.00 mL, 56.6 mmol, 9.0 equiv) dissolved in DCM (30 mL) was added dropwise in 1 h. The resulting mixture was warmed up to 25 °C and stirred overnight. The reaction solution was washed with saturated NaHCO_3_ solution three times, dried over MgSO_4_, and concentrated to give a crude product as dark orange oil. The product was recrystallized from hot ethanol as light yellow crystalline solid, which was further purified by column chromatography to afford white crystalline solid (3.28 g, 45.4%). The successful synthesis of initiator was confirmed by ^1^H NMR spectrum (Figure S1 in supplementary materials).

### 2.3. Synthesis of 4-Benzoylphenyl Methacrylate (BPMA)

BPMA was easily synthesized by esterifying 4-hydroxybenzophenone with methacroyl chloride. 4-Hydroxybenzophenone (10.5 g, 53.1 mmol, 1.0 equiv), TEA (15 mL, 106.2 mmol, 2.0 equiv), and DCM (150 mL) were added to a flask and was cooled to 0 °C by ice-bath followed by dropwise addition of methacroyl chloride (8.1 mL, 79.6 mmol, 1.5 equiv) solution in DCM (20 mL). After being warmed up to 25 °C and stirred for 12 h, the reaction mixture was washed with saturated aqueous NaHCO_3_ solution twice, dried over MgSO_4_, and concentrated in vacuum. The crude product was purified by recrystallization in ethanol three times to give pure product (10.2 g, 72.1%). The structure was revealed by ^1^H and ^13^C NMR spectra (Figure S2 in supplementary materials).

### 2.4. Synthesis of 2-(4-Benzoylphenoxy) Ethyl Methacrylate (BPOEMA) 

BPOEMA was synthesized by functionalizing 4-hydroxybenzophenone with 2-bromoethanol followed by esterification with methacryloyl chloride. 4-Hydroxybenzophenone (10.0 g, 50.44 mmol), KI (3.98 g, 24 mmol), K_2_CO_3_ (13.8 g, 100 mmol) and 2-bromoethanol (5.5 mL, 74 mmol) were dissolved in fresh dry DMF (150 mL) and stirred at 65 °C. Twelve hours later, extra K_2_CO_3_ (3.5 g, 25 mmol) and 2-bromoethanol (2.2 mL, 30 mmol) were added due to non-quantitative conversion revealed by thin-layer chromatography (TLC). This solution was stirring for further 12 h to improve the conversion. The reaction mixture was poured into 1 L of cold water. After filtering, colorless shiny platelets of product were collected and dried in a vacuum at 35 °C to give 9.2 g of product of 2-(4-benzoylphenoxy) ethanol (yield 73.9%), which was confirmed by ^1^H and ^13^C NMR spectra (Figure S3 in supplementary materials).

A solution of the as-synthesized 2-(4-benzoylphenoxy) ethanol (10.0 g, 41.2 mmol, 1.0 equiv) and TEA (12 mL, 82.5 mmol, 2.0 equiv) in DCM (150 mL) was transferred to a flask, and was cooled to 0 °C. After dropwise addition of methacryloyl chloride (6.3 mL, 62.2 mmol, 1.5 equiv) solution in DCM (20 mL), the reaction mixture was warmed up to 25 °C and was stirred for 24 h. The organic phase was washed with saturated aqueous NaHCO_3_ solution, dried over MgSO_4_, and was purified by column chromatography to give 8.7 g of BPOEMA as colorless viscous liquid (yield: 69.6%). The structure of BPOEMA was confirmed by ^1^H and ^13^C NMR spectra (Figure S4 in supplementary materials).

### 2.5. Protection of Hydroxyl Group of 2-Hydroxyethyl Methacrylate (HEMA)

HEMA (13 g, 1.0 equiv) and imidazole (17 g, 2.5 equiv) were dissolved in 25 mL of DCM and were cooled to 0 °C followed by slow addition of TBDMSCl (18 g, 1.2 equiv) solution in 10 mL of DCM. After warming up to 25 °C and stirring for 5 h, the reaction solution was washed with distilled water for three times, dried over MgSO_4_, concentrated and purified by column chromatography to afford 23.1 g of ProHEMA, which was revealed by ^1^H and ^13^C NMR (yield 95%) (Figure S5 in supplementary materials).

### 2.6. Synthesis of Star-Shaped Block Copolymers

The star-shaped block copolymers were synthesized through ATRP by a “grafting from” strategy. Taking the synthesis of P(BPMA-*co*-ProHEMA)-*b*-PMMA as an example, hexafunctional initiator (30 mg, 0.026 mmol), BPMA (3.127 g, 11.8 mmol), ProHEMA (5.74 g, 23.6 mmol), CuBr (22.5 mg, 0.157 mmol), and anisole (5.8 mL) were added to a Schlenk flask followed by three freeze–pump–thaw cycles to degas. After charging with Ar and addition of PMDETA (65 µL, 0.314 mmol), the solution was stirred at 70 °C for 40 min. After this reaction, the solution was quenched by putting the flask into an ice-bath and exposing to air. After 10 mL of THF was added, the solution was passed through a small silica gel column to remove the catalyst. The product was precipitated into methanol to get P(BPMA-*co*-ProHEMA), which was confirmed by ^1^H NMR spectrum and GPC traces.

The as-prepared P(BPMA-*co*-ProHEMA) (290 mg), MMA (800 mg, 8 mmol), CuBr (7.5 mg, 0.052 mmol), anisole (1.2 mL) were added to a Schlenk flask, and the solution was degassed by three freeze–pump–thaw cycles, followed by charging with Ar. After PMDETA (22 µL, 0.104 mmol) was added, the solution was stirred at 50 °C for 70 min. The reaction was processed according to the procedure used for the synthesis of P(BPMA-*co*-ProHEMA), as mentioned above, to give P(BPMA-*co*-ProHEMA)-*b*-PMMA, which was confirmed by ^1^H NMR spectrum and GPC traces.

The other two star-shaped block copolymers of P(BPMA-*co*-MMA)-*b*-P(ProHEMA) and P(BPOEMA-*co*-ProHEMA)-*b*-PMMA were synthesized in a similar way.

### 2.7. Synthesis of Fluorescent Organic Nanogels by McMurry Coupling Reaction

Freshly distilled THF (45 mL), zinc powder (1.5 g), and pyridine (1 mL) were added into a flask. The solution was degassed by bubbling Ar for 30 min and was cooled down to −78 °C by using a mixture of acetone and liquid nitrogen. To the solution, TiCl_4_ (1.2 mL) was added dropwise, followed by the addition of 5 mL of P(BPMA-*co*-ProHEMA)-*b*-MMA (200 mg) solution in freshly distilled THF. The resultant solution was warmed up to room temperature followed by refluxing in Ar atmosphere at 80 °C for 10 h. Twenty milliliters of K_2_CO_3_ aqueous solution (10%, weight percent) was added to quench the reaction. After 100 mL of THF was added and stirred for 30 min, the solution was filtered and concentrated in a vacuum, and precipitated into excess methanol to give the fluorescent nanogel.

### 2.8. Fabrication of Fluorescent Film by Mixing the Nanogel with PMMA

PMMA (100 mg) (*M*_w_ = 1.6 × 10^5^ g/mol, *Đ* = 1.97) (*Đ*: molar mass dispersity () and P(BPMA-*co*-ProHEMA)_gel_-*b*-PMMA (0.5 mg) were dissolved in 1 mL of THF. The solution was painted on a clean smooth glass, and was dried naturally to afford a fluorescent film. In addition, a pure PMMA film was prepared in the same way for comparison.

## 3. Results and Discussion

**Scheme 1:**
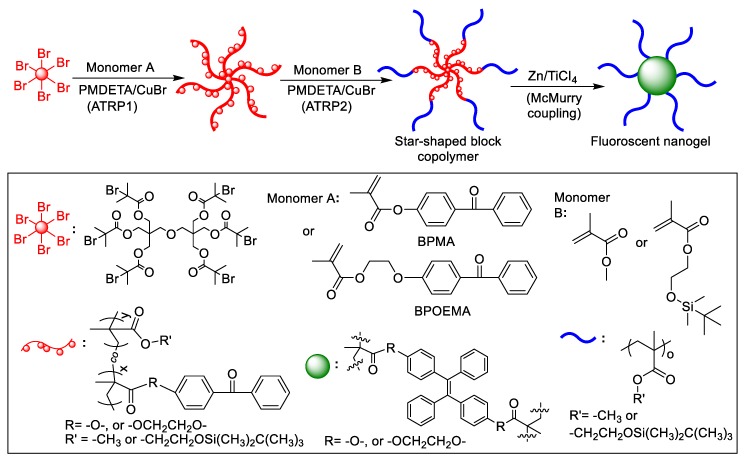
Synthesis of fluorescent organic nanogel by using a star-shaped block copolymer as precursor.

The fluorescent organic nanogel is achieved by using a polymeric precursor strategy. In this strategy, precise synthesis of a core–shell star-shaped block copolymer with crosslinkable potential AIEgen groups, pending on the inner blocks, were synthesized through ATRP by using a hexafunctional initiator. The pendent groups were crosslinked to generate AIE-active spacers, leading to a crosslinked network in the core, and thus, a fluorescent nanogel (Scheme 1).

### 3.1. Precise Synthesis Star-Shaped Block Copolymers by ATRP with a Hexafunctional Initiator

**Figure 1:**
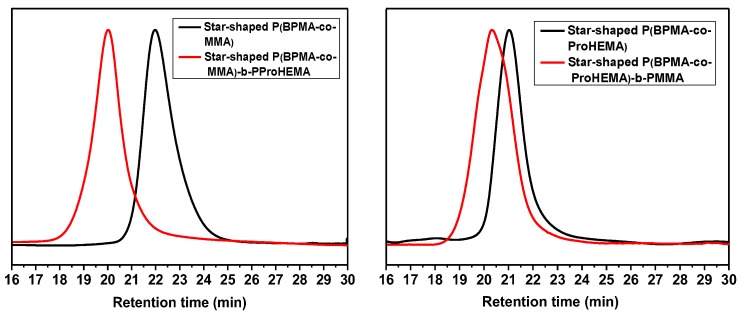
Synthesis of star-shaped block copolymers monitored by GPC traces (RI signal) (reaction conditions, molar ratio).

**Figure 2:**
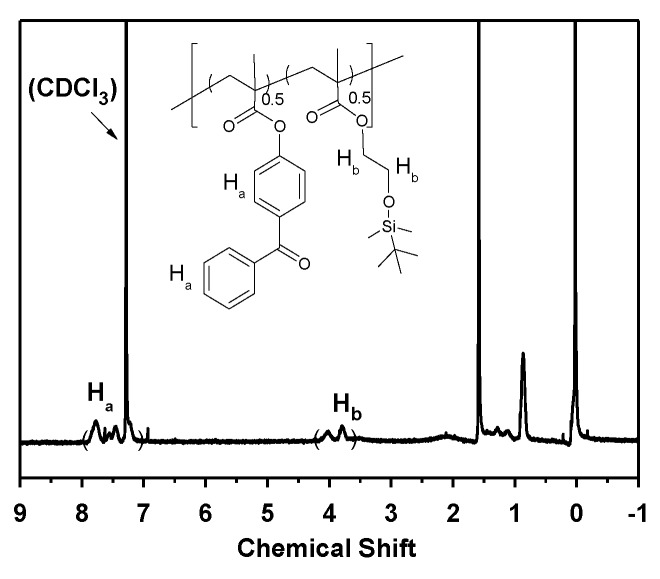
^1^H NMR spectrum of P(BPMA-*co*-ProHEMA) (400 Hz, CDCl_3_).

Starting from a hexafunctional initiator, star-shaped block copolymers were synthesized by using a “grafting from” strategy through ATRP. Taking the synthesis of P(BPMA-*co*-MMA)-*b*-P(ProHEMA) and P(BPMA-*co*-ProHEMA)-*b*-PMMA as examples (Figure 1), the pre-synthesized BPMA was copolymerized with MMA and ProHEMA, resulting in the responding star-shaped random copolymers of P(BPMA-*co*-MMA) and P(BPMA-co-ProHEMA), which served as macroinitiators for the followed ATRP of ProHEMA and MMA, leading to core–shell star-shaped block copolymer of P(BPMA-*co*-MMA)-*b*-P(ProHEMA) and P(BPOEMA-*co*-ProHEMA)-*b*-PMMA, respectively. The synthesis of the star-shaped copolymer was monitored by GPC traces (Figure 1). Both of the two star-shaped copolymers of P(BPMA-*co*-ProHEMA) and P(BPMA-*co*-MMA) showed narrow mono-distribution in GPC trace (refractive index (RI) signal). The *Đ*s of 1.17 and 1.16 were calculated by using a conventional calibration method built by using PS standards. In the case of P(BPMA-*co*-ProHEMA), the weight-averaged molecular weight (*M*_w_) of 6.36 × 10^4^ g/mol was determined by using static light scattering techniques performed on a MALLS detector equipped on the GPC system with a *dn/dc* value of 0.153 mL/g pre-determined on a RI detector. The compositions of PBPMA and PProHEMA (50% to 50% in mole) were revealed by the area ratio of protons in –*COOCH_2_CH_2_O*– of PProHEMA and the aromatic protons belonging to PBPMA in ^1^H NMR spectrum (Figure 2). Although the chemical shifts of aromatic protons in PBPMA and PBOEPMA (δ = 7.2–7.7 ppm) were overlapped with that of solvent (CDCl_3_) (δ = 7.26 ppm), the composition of the inner block can be calculated with a small deviation (2–3%) by eliminating the peak area of CDCl_3_ during integration (Figure S6, supplementary materials).

The resultant star-shaped polymer with an initiator moiety in each chain end served as hexafunctional macroinitiator for the following ATRP of MMA to afford the star-shaped block copolymer of P(BPMA-*co*-ProHEMA)-*b*-PMMA with an increase in molecular weight and *Đ* ,which was from 6.36 × 10^4^ to 1.70 × 10^5^ g/mol and from 1.17 to 1.28, respectively. By using the same method, the other two star-shaped block copolymers were also synthesized (Table 1). A shoulder that appeared in the P(BPMA-*co*-ProHEMA)-*b*-PMMA spectrum was attributed to the non-quantitative initiation of the star-shaped macroinitiator of P(BPMA-*co*-ProHEMA). ^1^H NMR spectra also indicated the successful synthesis of the products (Figures S6–S8 in supplementary materials).

### 3.2. Synthesis of Fluorescent Nanogels by Crosslinking the Inner Blocks through McMurry Reaction of the Pendent Benzophenone Groups

**Scheme 2:**
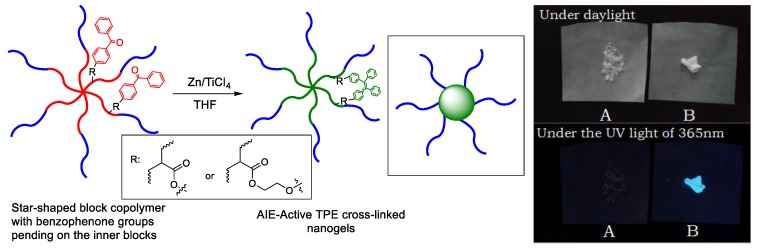
Gelation of the inner block of PBPMA or PBPOEMA through McMurry coupling reaction of pendent benzophenone groups and blue color emission of P(BPMA-*co*-ProHEMA)-*b*-PMMA (A) and responding nanogel (B).

**Figure 3:**
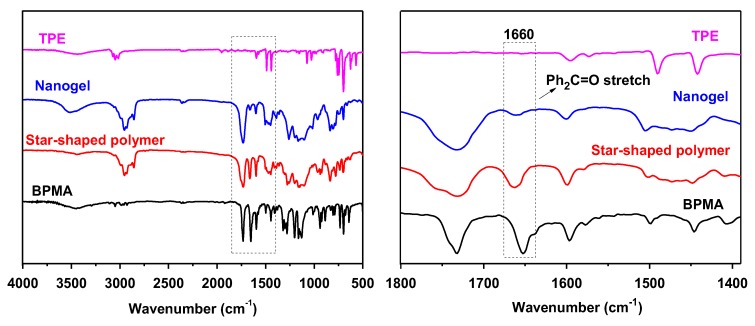
Successful synthesis of P(BPMA-*co*-ProHEMA)_gel_-*b*-PMMA confirmed by its FTIR spectrum compared with those of TPE, P(BPMA-*co*-ProHEMA)-*b*-PMMA and BPMA (left: full spectra, right: zoomed spectra).

The pendent benzophenone groups on the inner block of PBPMA or PBPOEMA in the star-shaped block copolymers are ready to be coupled through McMurry reaction in the presence of Zn/TiCl_4_, leading to the formation of TPE groups, and thus, gelation of the core (Scheme 2). In the gelation process, peripheral blocks of PMMA or PProHEMA are used to isolate the inner blocks and to suppress the intermolecular coupling. As a typical AIE-active fluorophore, the TPE groups serving as spacers in the polymeric network are fixed, and are strongly emissive, due to the highly restricted inter- and intramolecular movement by the polymeric network, taking the gelation of P(BPMA-*co*-ProHEMA)_gel_-*b*-PMMA as an example. After McMurry reaction, a strong blue light emission was observed, in contrast to its non-emissive precursor, indicating the formation of the TPE groups, and thus, the nanogel (images shown in Scheme 2). The successful coupling reaction was also proved by comparing FTIR spectra of TPE, P(BPMA-*co*-ProHEMA)_gel_-*b*-PMMA, P(BPMA-*co*-ProHEMA)-*b*-PMMA and BPMA (Figure 3). After McMurry reaction, the intensity of characteristic absorption peak at 1660 cm^−1^ of the nanogel (blue curve in Figure 3), attributed to the stretch of C=O bond in the benzophenone moiety, was significantly decreased, indicating the transformation from benzophenone groups to TPE groups, which had no absorption at this range. The residue of the peak at 1660 cm^−1^ indicated non-quantitative consumption of benzophenone groups in the McMurry reaction caused by higher and higher steric hindrance in the gelation process. Although the occurrence of McMurry reaction was qualitatively proved by the FTIR results and the fluorescence of the resultant nanogel, however, it is challenging to quantitatively estimate the efficiency. By using a similar method, another two fluorescent nanogels of P(BPMA-*co*-MMA)_gel_-*b*-P(ProHEMA) and P(BPOEMA-*co*-ProHEMA)_gel_-*b*-PMMA have also been successfully synthesized (Table 1).

### 3.3. The Fluorescent Behaviors and Sizes of the Organic Nanogels

**Figure 4:**
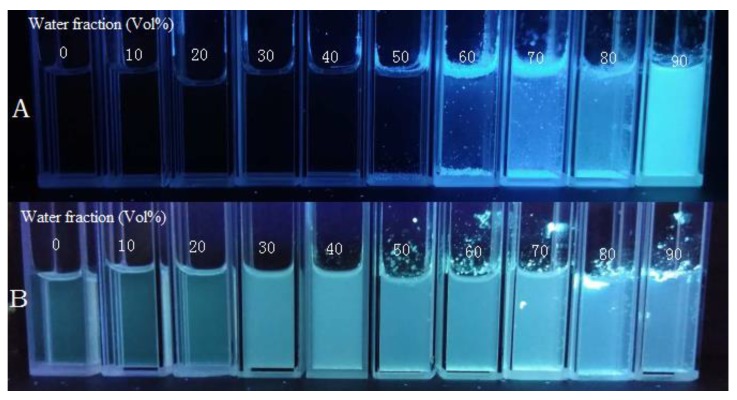
Comparison of fluorescence behaviors of (**A**) TPE (c_TPE_ = 2 mg/mL, 6.0 μmol/mL) with (**B**) nanogel of P(BPMA-*co*-ProHEMA)_gel_-*b*-PMMA (c_nanogel_ = 1 mg/mL, c_TPE unit_ < 0.37 μmol/mL) in THF/water mixed solvents with different water fractions from 0% to 90%. UV light (λ = 365 nm) was used to excite emission.

The regular small AIEgen molecules are not emissive in a dilute solution, but are strongly emissive at solid or aggregated state, due to the restricted intermolecular movement. In comparison, in the nanogel, the TPE moieties as spacers are covalently fixed in the polymeric network, leading to a highly restricted inter- and intramolecular movability, and high local concentration, resulting in strong emission in solute state or even in a high dilute solution, in addition to the solid and aggregated state. A comparison between fluorescent behaviors of the nanogels and TPE molecules were shown in Figure 4 and S9. Both nanogels and TPE were well dissolved in THF, to which water as poor solvent was added to prompt the aggregation. To enhance the contrast, a more diluted nanogel solution (1 mg/mL, *c*_TPE unit_ < 0.37 μmol/mL) was compared with TPE solution (2 mg/mL, 6.0 μmol/mL). Seen from Figure 4, the TPE was not emissive in THF solution, even with higher concentration than nanogel, until the water volume fractional ratio was up to 50%. By contrast, the nanogels emitted in pure THF and the emission was slightly enhanced by addition of water, which caused collapse of the nanogels, leading to further suppressed movability of TPE spacers in the nanogels, and thus, stronger emission.

**Figure 5:**
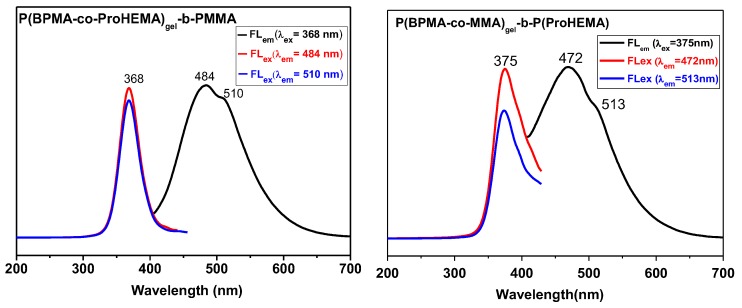
The fluorescent behaviors of P(BPMA-*co*-ProHEMA)_gel_-*b*-PMMA and P(BPMA-*co*-MMA)_gel_-*b*-P(ProHEMA).

Further fluorescence results revealed that P(BPMA-*co*-ProHEMA)_gel_-*b*-PMMA and P(BPMA-*co*-MMA)_gel_-*b*-P(ProHEMA) emitted at 484 and 472 nm with a shoulder at 510 and 513 nm, respectively (Figure 5). In the case of P(BPMA-*co*-ProHEMA)_gel_-*b*-PMMA, both of the two emissions have the same max exciting wavelength at 368 nm. The asymmetric emission peaks were found in all the as-prepared nanogels, and were supposed to be a consequence of the cis-trans isomerization of the TPE unit in the formation, which has been observed in the small molecule of TPE derivatives [63]. A quantum yield of P(BPMA-*co*-ProHEMA)_gel_-*b*-PMMA in THF solution (1 mg/mL) of 47.2% was determined (Table 1).

**Figure 6:**
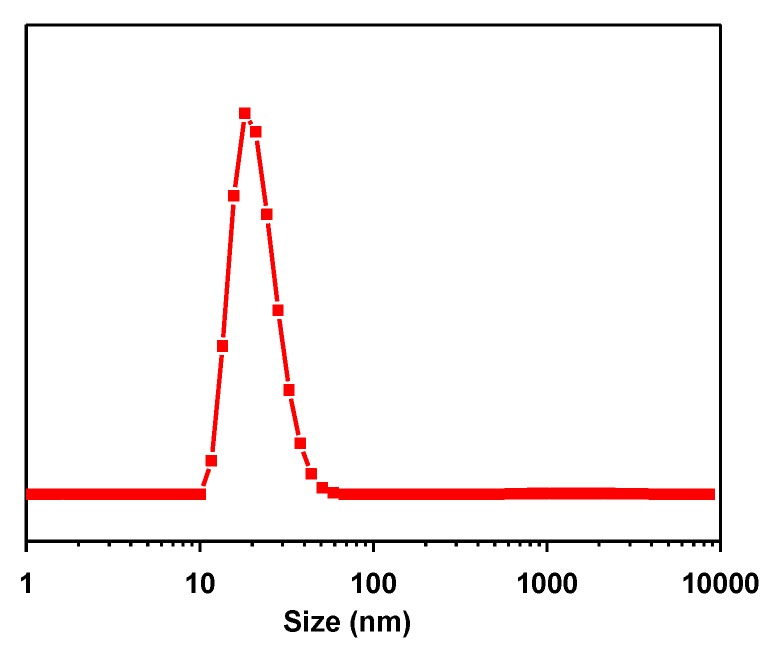
Hydrodynamic diameter of P(BPMA-*co*-ProHEMA)_gel_-*b*-PMMA in THF (0.2 mg/mL) calculated from dynamic light scattering result.

The size of nanogel plays an important role in their application. Hydrodynamic radiuses (*R*_h_) of three nanogels in their THF solution were determined by using dynamic light scattering technique (DLS). Taking the nanogels of P(BPMA-*co*-ProHEMA)_gel_-*b*-PMMA for example, the DLS result revealed a mono-distribution with a *D*_h_ of 21.4 nm (Figure 6). The theoretical averaged diameter of these nanogels of 54.6 nm calculated according to the degree of polymerization is larger than and the experimental value of 21.4 nm, which is supposed to be a result of high flexibility of the peripheral arms of the nanogel in solution, and collapse of the inner block caused by gelation. Similar results were also found in the other two samples (Table 1 and Figure S10 in supplementary materials).

**Figure 7:**
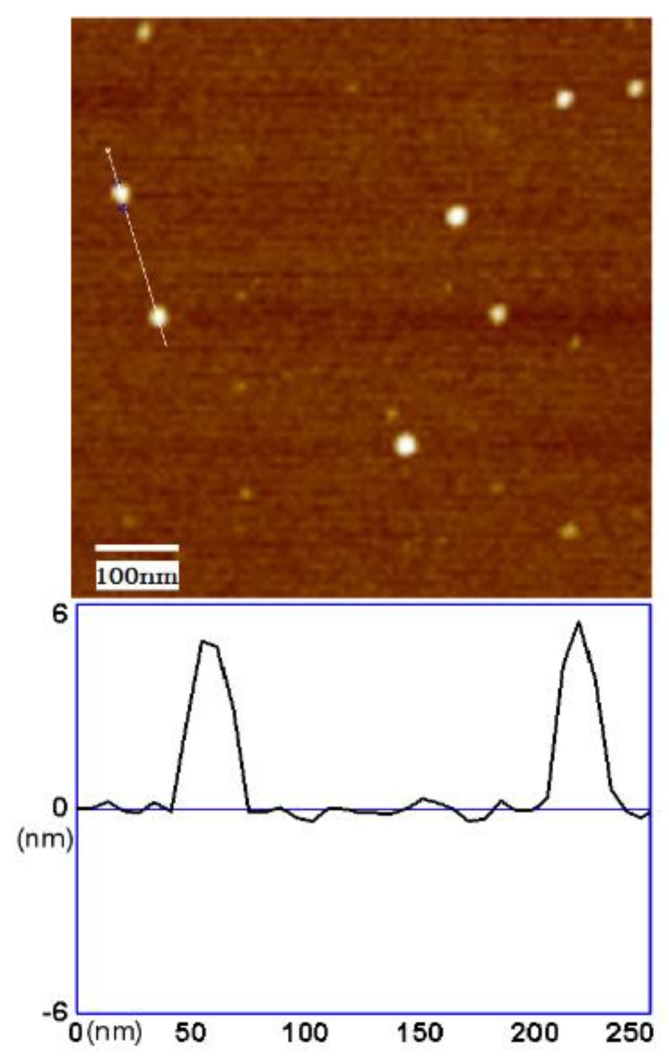
Structure of P(BPMA-*co*-ProHEMA)_gel_-*b*-PMMA on a silica wafer visualized by using AFM.

In addition, the structures of the nanogels on a silica substrate were visualized by using AFM. Seen from Figure 7, the nanogels of P(BPMA-*co*-ProHEMA)_gel_-*b*-PMMA adopt a spherical structure with a diameter around 30 nm. The larger size than its *D*_h_, revealed by DLS in dilute solution, was attributed to the collapse effect of the soft nanogel on the substrate indicated by a height of ~5 nm. The much smaller particles were attributed to the impurities of silicon wafer as used, which was confirmed by a control experiment.

### 3.4. Fabrication of a Fluorescent Film by Mixing the P(BPMA-co-ProHEMA)_gel_-b-PMMA

**Figure 8:**
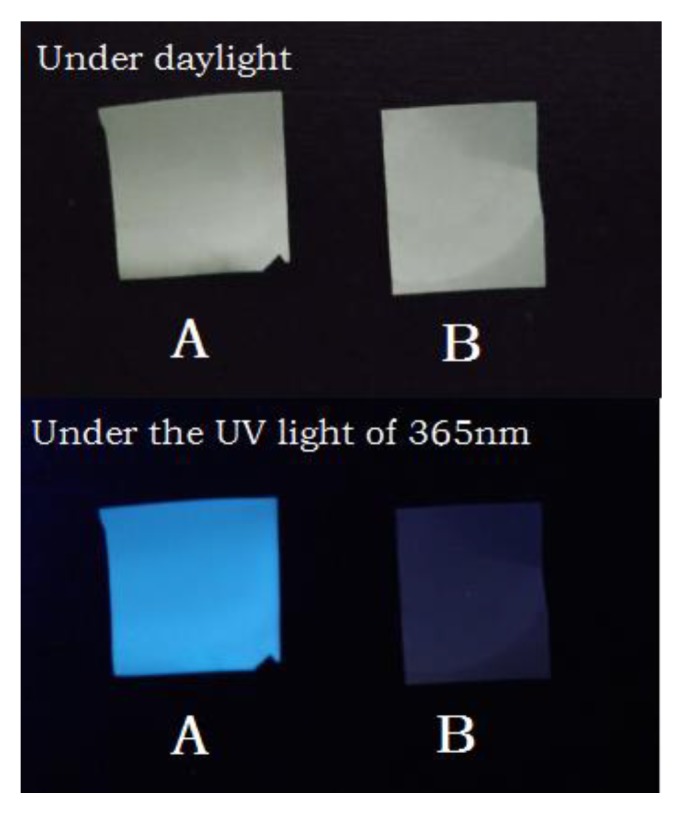
Fluorescent PMMA films prepared by mixing nanogel of P(BPMA-*co*-ProHEMA)gel-*b*-PMMA (0.5% weight percent) with PMMA.

Benefiting from the core–shell structure of the fluorescent nanogel, the peripheral block promised tunable compatibility with the other polymeric materials, by which the nanogel could be used as a macromolecular additive to fabricate fluorescent film. Taking the nanogel of P(BPMA-*co*-ProHEMA)_gel_-*b*-PMMA for example, a fluorescent PMMA film was fabricated by mixing the nanogel with PMMA homopolymer (*M*_w_ = 1.6 × 10^5^ g/mol, *Đ* = 1.97). Seen from Figure 8, the resultant film is strongly emissive, despite that the weight ratio of nanogel is as low as 0.5% in UV light (365 nm). Due to the good compatibility of the peripheral PMMA block with the PMMA homopolymer, the nanogels could be steadily dispersed in the PMMA, in contrast to the small fluorophore molecules which may move to the surface due to their poor compatibility and high movability in the film. In addition, the nanogels can be reasonably expected as a universal additive to the other polymers by choosing a proper peripheral block.

## 4. Conclusions

An easy and efficient approach to synthesize fluorescent nanogels has been successfully developed by using a pre-synthesized star-shaped polymer as precursor. The high universal and efficiency of this strategy have been proved by a series of fluorescent nanogels with tunable structures. Benefiting from the good controllability of ATRP over molecular weight and distribution, the star-shaped block copolymers can be precisely synthesized, and thus, promises a facile method to control the structures of the nanogels. The AIE-active spacer generated during the crosslinking of the inner blocks for constructing the polymeric network is an ideal fluorophore, due to the restricted structure in the resultant nanogels, and showed strong emission at both solute and aggregated states. The peripheral block is not only used to suppress the intermolecular crosslinking, but also used to improve the solubility and further functionalization. The design and synthesis of red-emissive fluorescent nanogels by using proper AIE-active spacer are ongoing.

## Figures and Tables

**Table 1:** Molecular characteristics of star-shaped block copolymers and resulting nanogels.

Star-shaped Blcok Copolymer	Star-shaped PA			Star-shaped PA-*b*-PB			PA_gel_-*b*-PB			QY_sol_. ^f^ (%)
	*M*_w_^a^ (kg/mol)	*Đ* ^b^	*dn/dc*^c^ (mL/g)	*M_w_*^a^ (kg/mol)	*Đ* ^b^	*dn/dc*^c^ (mL/g)	*D*_h_^d^ (nm)/PDI	λ_ex, max_^e^ (nm)	λ_em_^e^ (nm)	
P(BPMA_125_-*co*-ProHEMA_125_)-*b*-PMMA_1059_	63.60	1.17	0.153	169.50	1.28	0.112	21.4/0.43	368	484, 510	47.2
P(BPMA_113_-*co*-MMA_150_)-*b*-P(ProHEMA)_511_	45.33	1.16	0.148	169.90	1.37	0.128	26.7/0.47	375	472, 513	- ^g^
P(BPOEMA_275_-*co*-ProHEMA_559_)-*b*-PMMA_496_	221.9	1.22	0.121	271.50	1.23	0.110	26.8/0.28	381	478, 513	- ^g^

^a^ The M_w_s were calculated from static light scattering results determined by a GPC-MALLS system; ^b^ *Đ*s were determined by GPC (RI signal trace) calibrated by using polystyrene standards; ^c^ *dn/dc* values were determined by using a differential refractometer; ^d^ hydrodynamic diamater (*D*_h_) and polydispersity index (PDI) were determined by using dynamic light scattering technique; ^e^ the max exciting and emission wavelengths nanogels in THF were determined by using a fluorescence spectrophotometer; ^f^ QY_sol_ (1 mg/mL in THF) is determined by using a spectrofluorometer; ^g^ not determined.

## Supplementary Materials

Supplementary Materials are available online at http://www.mdpi.com/2073-4360/10/7/722/s1, Figure S1: ^1^H NMR spectrum of hexafunctional initiator; Figure S2: ^1^H and ^13^C NMR spectra of BPMA; Figure S3: ^1^H and ^13^C NMR spectra of 2-(4-benzoylphenoxy) ethanol, Figure S4: ^1^H and ^13^C NMR spectra of BPOEMA; Figure S5: ^1^H and ^13^C NMR spectra of ProHEMA; Figure S6: ^1^H NMR spectrum of P(BPMA-*co*-ProHEMA)-*b*-PMMA; Figure S7: ^1^H NMR spectrum of P(BPMA-*co*-MMA)-*b*-P(ProHEMA); Figure S8: ^1^H NMR spectrum of P(BPOEMA-*co*-ProHEMA)-*b*-PMMA; Figure S9: The fluorescent characteristics of organic nanogels in THF/water mixture solvents; Figure S10: Hydrodynamic diameter of P(BPMA-*co*-MMA)_gel_-*b*-ProHEMA and P(BPOEMA-*co*-ProHEMA)_gel_-*b*-PMMA in THF calculated from dynamic light scattering results.

## References

[1] Peng H.S., Chiu D.T. (2015). Soft fluorescent nanomaterials for biological and biomedical imaging. Chem. Soc. Rev..

[2] Wolfbeis O.S. (2015). An overview of nanoparticles commonly used in fluorescent bioimaging. Chem. Soc. Rev..

[3] Chernov K.G., Redchuk T.A., Omelina E.S., Verkhushaa V.V. (2017). Near-Infrared Fluorescent Proteins, Biosensors, and Optogenetic Tools Engineered from Phytochromes. Chem. Rev..

[4] Acharya A., Bogdanov A.M., Grigorenko B.L., Bravaya K.B., Nemukhin A.V., Lukyanov K.A., Krylov A.I. (2017). Photoinduced Chemistry in Fluorescent Proteins: Curse or Blessing?. Chem. Rev..

[5] Ma X., Shi X., Bai S., Zhang J., Hou M., Zhang T., Li B.S., Xue P., Kang Y., Xu Z. (2018). Water-soluble fluorescent unimolecular micelles: Ultra-small size, tunable fluorescence emission from the visible to NIR region and enhanced biocompatibility for in vitro and in vivo bioimaging. Chem. Commun..

[6] Yu G., Zhou X., Zhang Z., Han C., Mao Z., Gao C., Huang F. (2012). Pillar [6] arene/Paraquat Molecular Recognition in Water: High Binding Strength, pH-Responsiveness, and Application in Controllable Self-Assembly, Controlled Release, and Treatment of Paraquat Poisoning. J. Am. Chem. Soc..

[7] Zhang C., Jin S., Li S., Xue X., Liu J., Huang Y., Jiang Y., Chen W.Q., Zou G., Liang X.J. (2014). Imaging intracellular anticancer drug delivery by self-assembly micelles with aggregation-induced emission (AIE micelles). ACS Appl. Mater. Interfaces.

[8] Reisch A., Klymchenko A.S. (2016). Fluorescent Polymer Nanoparticles Based on Dyes: Seeking Brighter Tools for Bioimaging. Small.

[9] Cao L., Li X., Wang S., Li S., Li Y., Yang G. (2014). A novel nanogel-based fluorescent probe for ratiometric detection of intracellular pH values. Chem. Commun..

[10] Peng H.S., Stolwijk J.A., Sun L.N., Wegener J., Wolfbeis O.S. (2010). A nanogel for ratiometric fluorescent sensing of intracellular pH values. Angew. Chem..

[11] Uchiyama S., Tsuji T., Kawamoto K., Okano K., Fukatsu E., Noro T., Ikado K., Yamada S., Shibata Y., Hayashi T. (2018). A Cell-Targeted Non-Cytotoxic Fluorescent Nanogel Thermometer Created with an Imidazolium-Containing Cationic Radical Initiator. Angew. Chem..

[12] Gota C., Okabe K., Funatsu T., Harada Y., Uchiyama S. (2009). Hydrophilic fluorescent nanogel thermometer for intracellular thermometry. J. Am. Chem. Soc..

[13] Xing T., Mao C., Lai B., Yan L. (2012). Synthesis of disulfide-cross-linked polypeptide nanogel conjugated with a near-infrared fluorescence probe for direct imaging of reduction-induced drug release. ACS Appl. Mater. Interfaces.

[14] Li F., Bae B.C., Na K. (2010). Acetylated hyaluronic acid/photosensitizer conjugate for the preparation of nanogels with controllable phototoxicity: Synthesis, characterization, autophotoquenching properties, and in vitro phototoxicity against HeLa cells. Bioconj. Chem..

[15] Min K.I., Kim D.H., Lee H.J., Lin L., Kim D.P. (2018). Direct Synthesis of a Covalently Self-Assembled Peptide Nanogel from a Tyrosine-Rich Peptide Monomer and Its Biomineralized Hybrids. Angew. Chem. Int. Ed..

[16] Li H., Zhang X., Zhang X., Wang K., Liu H., Wei Y. (2015). Facile preparation of biocompatible and robust fluorescent polymeric nanoparticles via PEGylation and cross-linking. ACS Appl. Mater. Interfaces.

[17] Amamoto Y., Kikuchi M., Otsuka H., Takahara A. (2010). Arm-replaceable star-shaped nanogels: Arm detachment and arm exchange reactions by dynamic covalent exchanges of alkoxyamine units. Polym. J..

[18] Amamoto Y., Kikuchi M., Masunaga H., Sasaki S., Otsuka H., Takahara A. (2010). Intelligent Build-Up of Complementarily Reactive Diblock Copolymers via Dynamic Covalent Exchange toward Symmetrical and Miktoarm Star-shaped Nanogels. Macromolecules.

[19] Zhang Z., Yin L., Tu C., Song Z., Zhang Y., Xu Y., Tong R., Zhou Q., Ren J., Cheng J. (2013). Redox-Responsive, Core Cross-Linked Polyester Micelles. ACS Macro Lett..

[20] Atta A.M., Dyab A.K.F., Allohedan H.A. (2013). A novel route to prepare highly surface active nanogel particles based on nonaqueous emulsion polymerization. Polym. Adv. Technol..

[21] Cao Z., Ziener U. (2013). Synthesis of nanostructured materials in inverse miniemulsions and their applications. Nanoscale.

[22] Yuan Y.Y., Du J.Z., Song W.J., Wang F., Yang X.Z., Xiong M.H., Wang J. (2012). Biocompatible and functionalizable polyphosphate nanogel with a branched structure. J. Mater. Chem..

[23] Pang X., Zhao L., Han W., Xin X., Lin Z. (2013). A general and robust strategy for the synthesis of nearly monodisperse colloidal nanocrystals. Nat. Nanotechnol..

[24] Pang X., He Y., Jung J., Lin Z. (2016). 1D nanocrystals with precisely controlled dimensions, compositions, and architectures. Science.

[25] Huang K., Rzayev J. (2011). Charge and size selective molecular transport by amphiphilic organic nanotubes. J. Am. Chem. Soc..

[26] Feng C., Pang X., He Y., Li B., Lin Z. (2014). Robust Route to Unimolecular Core–Shell and Hollow Polymer Nanoparticles. Chem. Mater..

[27] Huang K., Rzayev J. (2009). Well-defined organic nanotubes from multicomponent bottlebrush copolymers. J. Am. Chem. Soc..

[28] Oh J.K., Siegwart D.J., Matyjaszewski K. (2007). Synthesis and biodegradation of nanogels as delivery carriers for carbohydrate drugs. Biomacromolecules.

[29] Beija M., Afonso C.A.M., Martinho J.M.G. (2009). Synthesis and applications of Rhodamine derivatives as fluorescent probes. Chem. Soc. Rev..

[30] Lavis L.D. (2017). Teaching Old Dyes New Tricks: Biological Probes Built from Fluoresceins and Rhodamines. Annu. Rev. Biochem..

[31] Duke R.M., Veale E.B., Pfeffer F.M., Kruger P.E., Gunnlaugsson T. (2010). Colorimetric and fluorescent anion sensors: An overview of recent developments in the use of 1,8-naphthalimide-based chemosensors. Chem. Soc. Rev..

[32] Grabchev I., Staneva D., Betcheva R. (2012). Fluorescent Dendrimers As Sensors for Biologically Important Metal Cations. Curr. Med. Chem..

[33] Klymchenko A.S. (2017). Solvatochromic and Fluorogenic Dyes as Environment-Sensitive Probes: Design and Biological Applications. Acc. Chem. Res..

[34] Guo Z., Park S., Yoon J., Shin I. (2014). Recent progress in the development of near-infrared fluorescent probes for bioimaging applications. Chem. Soc. Rev.,.

[35] Hong Y., Lam J.W.Y., Tang B.Z. (2009). Aggregation-induced emission: Phenomenon, mechanism and applications. Chem. Commun..

[36] Luo J., Xie Z., Lam J.W.Y., Cheng L., Chen H., Qiu C., Kwok H.S.K., Zhan X., Liu Y., Zhu D. (2001). Aggregation-induced emission of 1-methyl-1,2,3,4,5-pentaphenylsilole. Chem. Commun..

[37] Mei J., Leung N.L., Kwok R.T., Lam J.W.Y., Tang B.Z. (2015). Aggregation-Induced Emission: Together We Shine, United We Soar!. Chem. Rev..

[38] Zhan R., Pan Y., Manghnani P.N., Liu B. (2017). AIE Polymers: Synthesis, Properties, and Biological Applications. Macromol. Biosci..

[39] Li D., Yu J. (2016). AIEgens-Functionalized Inorganic-Organic Hybrid Materials: Fabrications and Applications. Small.

[40] Zhao W., Li C., Liu B., Wang X., Li P., Wang Y., Wu C., Yao C., Tang T., Liu X. (2014). A New Strategy To Access Polymers with Aggregation-Induced Emission Characteristics. Macromolecules.

[41] Dong R., Ravinathan S.P., Xue L., Li N., Zhang Y., Zhou L., Cao C., Zhu X. (2016). Dual-responsive aggregation-induced emission-active supramolecular nanoparticles for gene delivery and bioimaging. Chem. Commun..

[42] Liow S.S., Zhou H., Sugiarto S., Guo S., Chalasani M.L., Verma N.K., Xu J., Loh X.J. (2017). Highly Efficient Supramolecular Aggregation-Induced Emission-Active Pseudorotaxane Luminogen for Functional Bioimaging. Biomacromolecules.

[43] Zhu Z., Qian J., Zhao X., Qin W., Hu R., Zhang H., Li D., Xu Z., Tang B.Z., He S. (2016). Stable and Size-Tunable Aggregation-Induced Emission Nanoparticles Encapsulated with Nanographene Oxide and Applications in Three-Photon Fluorescence Bioimaging. ACS Nano.

[44] Ding D., Li K., Liu B., Tang B.Z. (2013). Bioprobes Based on AIE Fluorogens. Acc. Chem. Res..

[45] Mei J., Huang Y., Tian H. (2018). Progress and Trends in AIE-Based Bioprobes: A Brief Overview. ACS Appl. Mater. Interfaces.

[46] Wang Y.F., Zhang T., Liang X.J. (2016). Aggregation-Induced Emission: Lighting up Cells, Revealing Life!. Small.

[47] Shi J., Li Y., Li Q., Li Z. (2018). Enzyme-Responsive Bioprobes Based on the Mechanism of Aggregation-Induced Emission. ACS Appl. Mater. Interfaces.

[48] Gao M., Tang B.Z. (2017). Aggregation-induced emission probes for cancer theranostics. Drug Discov. Today.

[49] Gu X., Kwok R.T.K., Lam J.W.Y., Tang B.Z. (2017). AlEgens for biological process monitoring and disease theranostics. Biomaterials.

[50] Zhan C., You X., Zhang G., Zhang D. (2016). Bio-/Chemosensors and Imaging with Aggregation-Induced Emission Luminogens. Chem. Rec..

[51] Han T., Feng X., Tong B., Shi J., Chen L., Zhi J., Dong Y. (2012). A novel “turn-on” fluorescent chemosensor for the selective detection of Al^3+^ based on aggregation-induced emission. Chem. Commun..

[52] Xu X., Huang J., Li J., Yan J., Qin J., Li Z. (2011). A graphene oxide-based AIE biosensor with high selectivity toward bovine serum albumin. Chem. Commun..

[53] Gao M., Tang B.Z. (2017). Fluorescent Sensors Based on Aggregation-Induced Emission: Recent Advances and Perspectives. ACS Sens..

[54] Huang J., Sun N., Chen P., Tang R., Li Q., Ma D., Li Z. (2014). Largely blue-shifted emission through minor structural modifications: Molecular design, synthesis, aggregation-induced emission and deep-blue OLED application. Chem. Commun..

[55] Feng X.J., Peng J., Xu Z., Fang R., Zhang H.R., Xu X., Li L., Gao J., Wong M.S. (2015). AIE-Active Fluorene Derivatives for Solution-Processable Nondoped Blue Organic Light-Emitting Devices (OLEDs). ACS Appl. Mater. Interfaces.

[56] Islam A., Zhang D., Peng R., Yang R., Hong L., Song W., Wei Q., Duan L., Ge Z. (2017). Non-Doped Sky-Blue OLEDs Based on Simple Structured AIE Emitters with High Efficiencies at Low Driven Voltages. Chem. Asian J..

[57] Furue R., Nishimoto T., Park I.S., Lee J., Yasuda T. (2016). Aggregation-Induced Delayed Fluorescence Based on Donor/Acceptor-Tethered Janus Carborane Triads: Unique Photophysical Properties of Nondoped OLEDs. Angew. Chem..

[58] Zhang F., Fan J., Yu H., Ke Z., Nie C., Kuang D., Shao G., Su C. (2015). Nonplanar Organic Sensitizers Featuring a Tetraphenylethene Structure and Double Electron-Withdrawing Anchoring Groups. J. Org. Chem..

[59] Ong K.H., Liu B. (2017). Applications of Fluorogens with Rotor Structures in Solar Cells. Molecules.

[60] Li Y., Li Z., Ablekim T., Ren T., Dong W.J. (2014). Rational design of tetraphenylethylene-based luminescent down-shifting molecules: Photophysical studies and photovoltaic applications in a CdTe solar cell from small to large units. Phys. Chem. Chem. Phys..

[61] Li H., Zhang X., Zhang X., Yang B., Yang Y., Huang Z., Wei Y. (2014). Zwitterionic red fluorescent polymeric nanoparticles for cell imaging. Macromol. Biosci..

[62] Zhang Z., Bilalis P., Zhang H., Gnanou Y., Hadjichristidis N. (2017). Core Cross-Linked Multiarm Star Polymers with Aggregation-Induced Emission and Temperature Responsive Fluorescence Characteristics. Macromolecules.

[63] Wang J., Mei J., Hu R., Sun J.Z., Qin A., Tang B.Z. (2012). Click Synthesis, Aggregation-Induced Emission, E/Z Isomerization, Self-Organization, and Multiple Chromisms of Pure Stereoisomers of a Tetraphenylethene-Cored Luminogen. J. Am. Chem. Soc..

