# Supplementary material for: A Facile Approach towards Fluorescent Nanogels with AIE-Active Spacers
Source: Polymers (Basel). 2018 Jul 1;10(7):722. doi: 10.3390/polym10070722 (PMC6403691; doi:10.3390/polym10070722)
Supplement: Supplementary file 1 [file polymers-10-00722-s001.pdf]

Supporting information

A facile approach towards florescent nanogel with AIE-active spacers

Meiran Feng, Laiping Fang, Fujun Guan, Siying Huang, Yinwei Cheng, Yancui Liang and Hefeng Zhang*

Department of Chemistry and Key Laboratory for Preparation and Application of Ordered Structural Materials of Guangdong Province, Shantou University, Shantou, 515063, P. R. China.

* Correspondence: hfzhang@stu.edu.cn

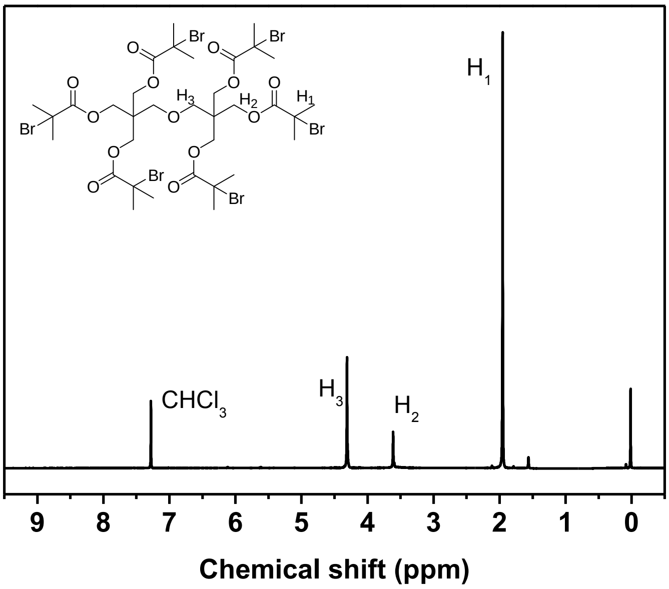

Figure S1. ^{1}H NMR spectrum of hexafunctional initiator (400 MHz, $CDCl_3$)

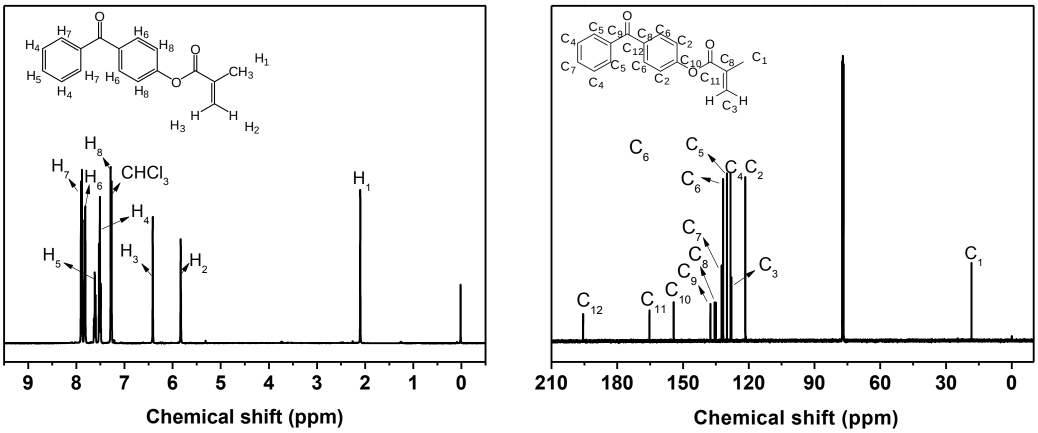

Figure S2. ^{1}H (left) and ^{13}C (left) NMR spectra of BPMA (400 MHz, $CDCl_3$)

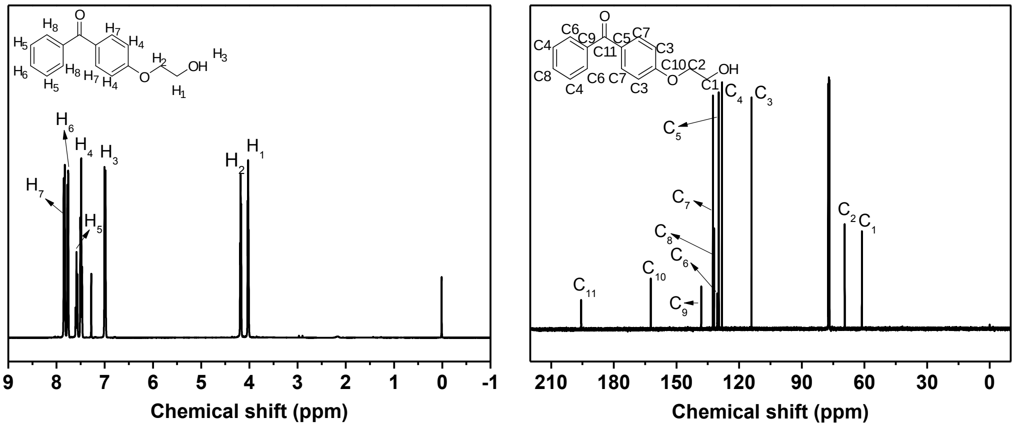

Figure S3. ^{1}H (left) and ^{13}C (right) NMR spectra of 2-(4-benzoylphenoxy) ethanol (400 MHz, $CDCl_3$)

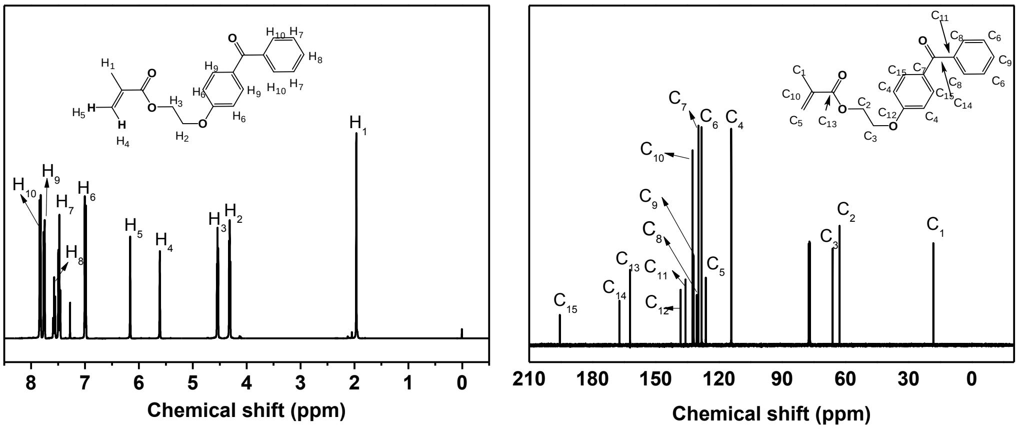

Figure S4. ^{1}H (left) and ^{13}C (right) NMR spectra of BPOEMA (400 MHz, $CDCl_3$)

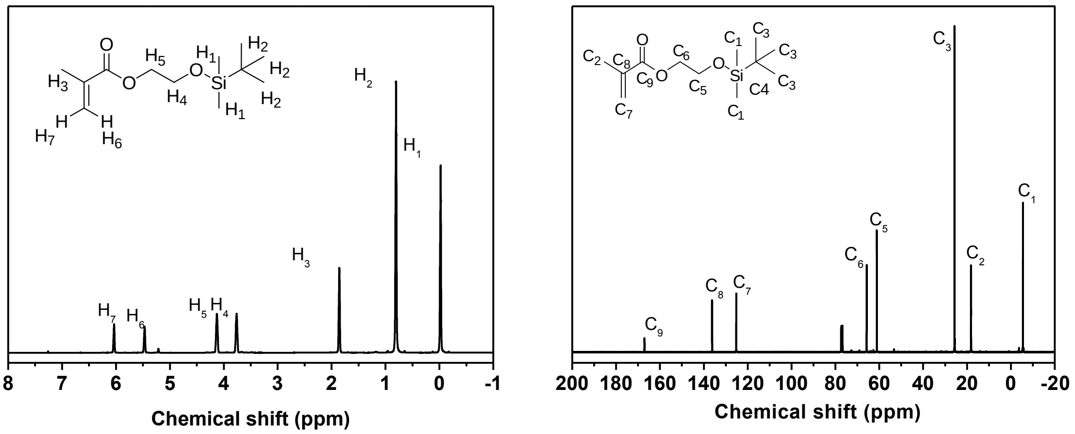

Figure S5. ^{1}H (left) and ^{13}C (right) NMR spectra of ProHEMA (400 MHz, $CDCl_3$)

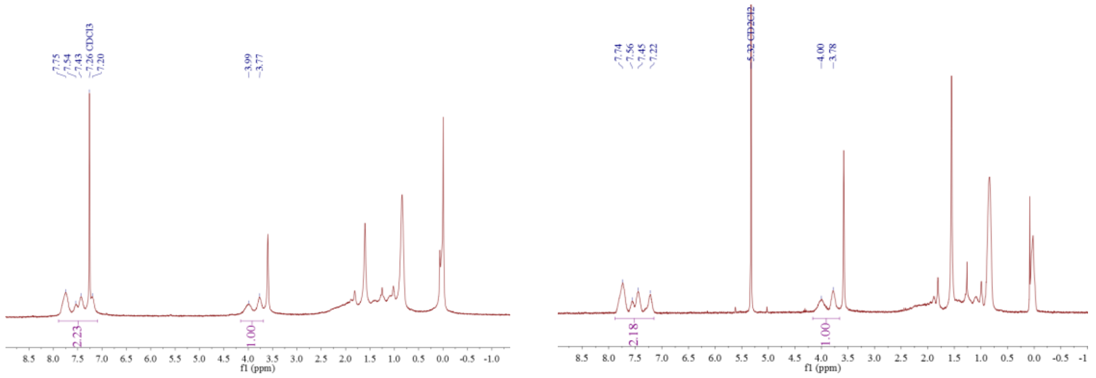

Figure S6. ^{1}H NMR spectrum of P(BPMA-*co*-ProHEMA)-*b*-PMMA in $CDCl_3$ (left) and $CDCl_2$ (right) (400 MHz). By eliminating the peak area of solvent ($CDCl_3$) (δ = 7.26 ppm) during integration, similar compositions were calculated with an acceptable deviation (~ 2%) (PBPMA : ProHEMA = 0.99:1 ($CDCl_3$) and 0.97:1 ($CDCl_2$)) although the chemical shifts of aromatic protons in PBPMA (δ = 7.2 - 7.7 ppm) overlapped with that of $CDCl_3$.

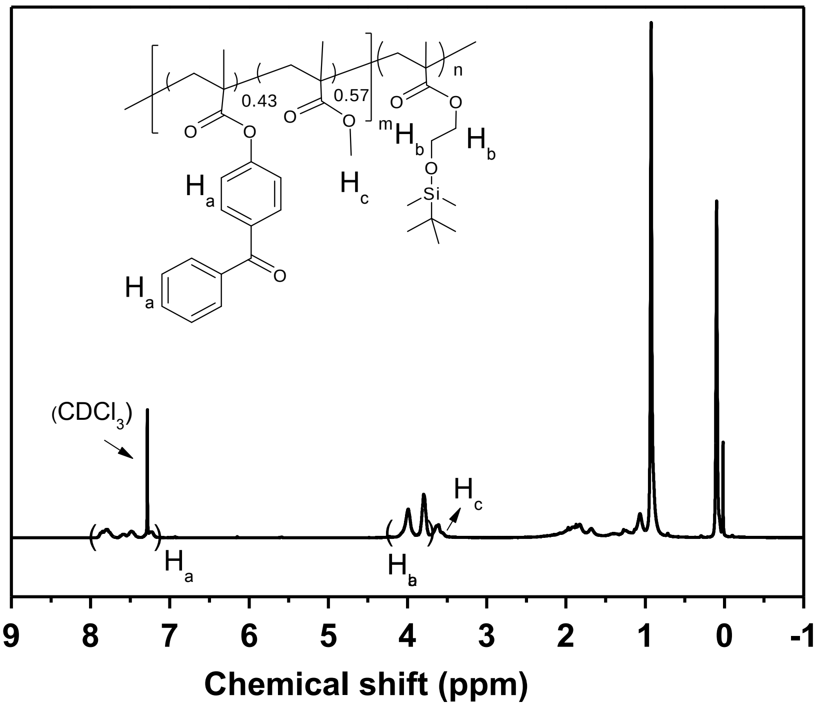

Figure S7. ^{1}H NMR spectrum of P(BPMA-*co*-MMA)-*b*-P(ProHEMA) (400 MHz, $CDCl_3$)

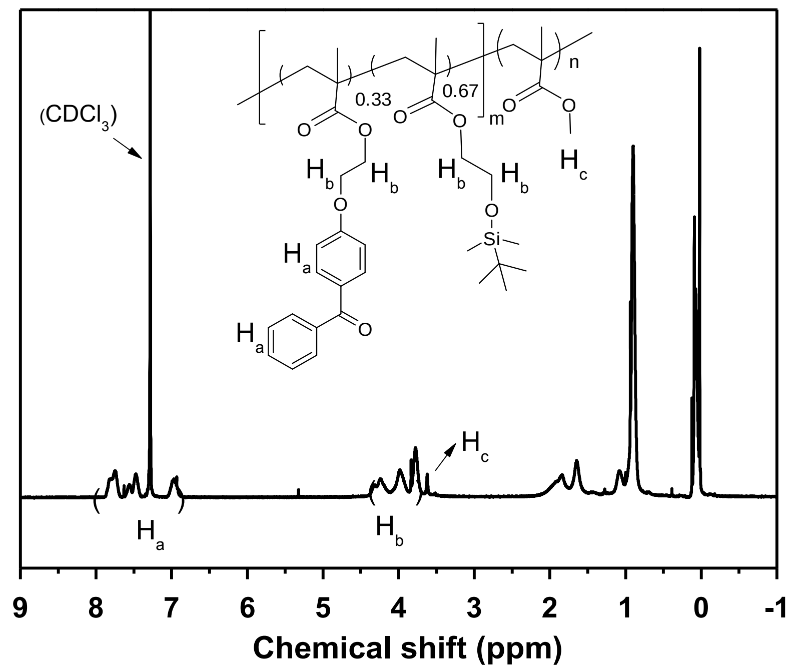

Figure S8. ${}^{1}H$ NMR spectrum of P(BPOEMA-*co*-ProHEMA)-*b*-PMMA (400 MHz, $CDCl_3$)

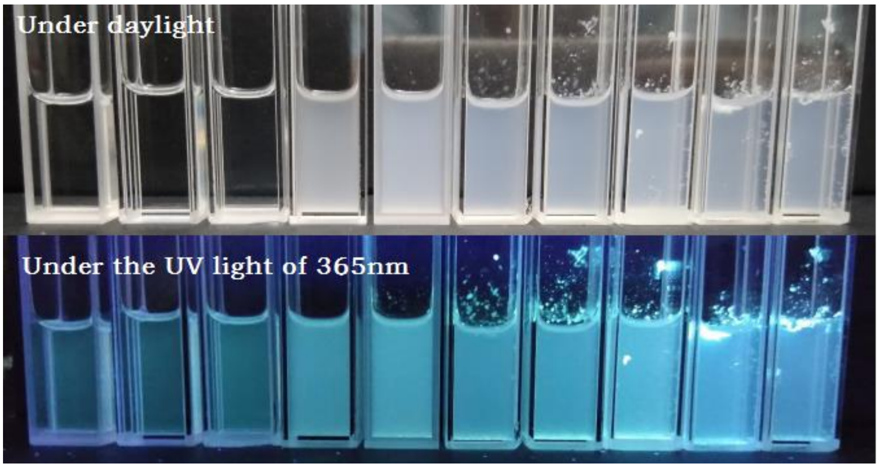

Figure S9. The fluorescent characteristics of P(BPMA-*co*-ProHEMA)$_{gel}$-*b*-MMA in THF/water mixture solvents (water fraction is 0% to 90% from left to right with a 10% increase).

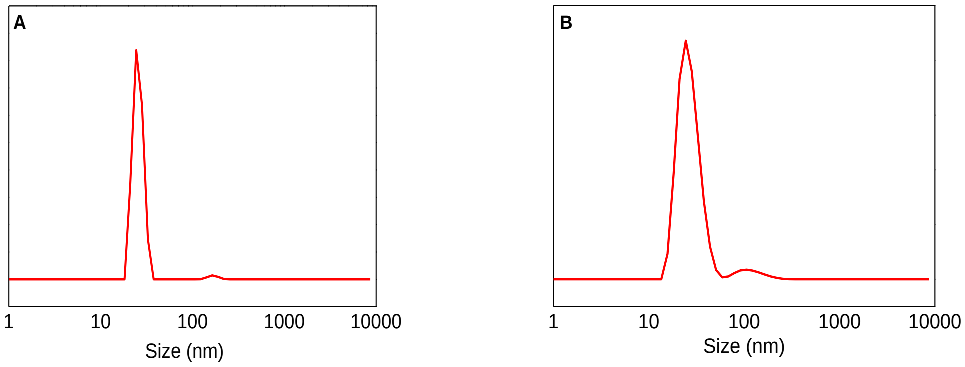

Figure S10. Hydrodynamic diameters of A: P(BPMA-*co*-MMA)$_{gel}$-*b*-ProHEMA and B: P(BPOEMA-*co*-ProHEMA)$_{gel}$-*b*-PMMA in THF calculated from dynamic light scattering results
